# Mitochondrial Dysfunctions: A Thread Sewing Together Alzheimer's Disease, Diabetes, and Obesity

**DOI:** 10.1155/2019/7210892

**Published:** 2019-06-16

**Authors:** Giulia Rigotto, Emy Basso

**Affiliations:** ^1^Department of Biomedical Sciences, University of Padua, 35131 Padua, Italy; ^2^National Research Council, Department of Biomedical Science, Neuroscience Institute (Padua Section), 35131 Padua, Italy

## Abstract

Metabolic disorders are severe and chronic impairments of the health of many people and represent a challenge for the society as a whole that has to deal with an ever-increasing number of affected individuals. Among common metabolic disorders are Alzheimer's disease, obesity, and type 2 diabetes. These disorders do not have a univocal genetic cause but rather can result from the interaction of multiple genes, lifestyle, and environmental factors. Mitochondrial alterations have emerged as a feature common to all these disorders, underlining perhaps an impaired coordination between cellular needs and mitochondrial responses that could contribute to their development and/or progression.

## 1. Introduction

Obesity, diabetes, and Alzheimer's disease (AD) are long-term pathologies affecting millions of individuals in the developed countries and progressively expanding also in developing countries. Moreover, obesity and diabetes have long been considered significant risk factors for the progression of late onset Alzheimer's disease, and in recent years, a growing number of epidemiological studies are finding compelling connections between these complex metabolic disorders [1, 2]. A coordinated approach to characterize common, shared traits would be helpful to better understand these conditions and, hopefully, devise prevention strategies. There are several important and recognized intersections among these disorders that point to common pathological developments, but also possibly common, impaired metabolic pathways. One such trait is insulin resistance which commonly occurs in peripheral tissues in obesity and diabetes, but has been also shown to occur in the brain of Alzheimer's disease patients [3, 4]. The canonical function of insulin is the regulation of body metabolism, but it has as well a role in promoting synaptic and neuronal plasticity which are greatly compromised in AD [5, 6]. Dysregulated lipid metabolism is common in obesity and diabetes; the increased levels of free fatty acids (FFA) in the bloodstream can inhibit the antilipolytic action of insulin and alter the permeability of the blood brain barrier (BBB) resulting in FFA infiltrating in the brain and contributing to brain damage and declining cognitive functions [7, 8].

Another common trait is the presence of advanced glycation end products (AGEs) found in both diabetic and AD patients [9, 10]; long standing, elevated glucose plasma levels promote the formation of covalent adducts with proteins through a nonenzymatic reaction called glycation. AGE products alter the structure and function of biological molecules and increase oxidative stress promoting intracellular damage and apoptosis [11]. AGE products bind to the multiligand transmembrane receptor for advanced glycation end products (RAGE), and this interaction results in the activation of downstream signaling pathways including the activation of NF-*κ*B transcription factor and the triggering of proinflammatory responses [12]. It has also been reported that RAGE promotes A*β* peptide production [13] with consequences on the integrity of the BBB [14].

Inflammation is another common feature of these disorders. Obese patients display low-grade chronic inflammation in the adipose tissue characterized by the infiltration of inflammatory cells and the increased production and secretion of proinflammatory factors [15, 16]. High levels of FFA introduced with the diet can trigger an inflammatory cascade initiated by Toll-like receptor 4 (TLR4) and the release of proinflammatory cytokines such as TNF-*α* and interleukins IL-1*β* and IL-6 [17]. The pathogenesis of diabetes is as well associated with high immune system activation [18], and inflammation is the main cause of diabetes complications such as retinopathy, nephropathy, and neuropathy. It is known that chronic hyperglycemia activates NF-*κ*B and stimulates the release of proinflammatory cytokines [19]. The effects of NF-*κ*B-mediated metabolic inflammation are deleterious and can result in impairments of intracellular signaling and disruptions of metabolic physiology. Neuroinflammation has been known for many years to occur in AD: glial cell activation is observed in the proximity of the lesions [20]; moreover, preclinical genetic data have shown that the activation of the immune system contributes to the pathogenesis of the disease [21–23].

Of course, the key issue is to understand how mostly peripheral metabolic impairments observed in obesity and diabetes might be connected with cerebral pathological alterations present in AD.

Mitochondria perform a number of essential activities for cellular/tissue differentiation and survival ranging from ATP production to reactive oxygen species (ROS) signaling, catabolism of fatty acid, ion homeostasis, biosynthesis of heme, and cell death. Because of this central role played in cell metabolism, it is not surprising that mitochondrial malfunctioning has been linked to various different metabolic disorders as different as type 2 diabetes [24], cancer [25], and neurodegenerative disorders [26, 27].

Mitochondrial dysfunction is a broad term that comprises maladaptive physiological responses such as impaired substrate catabolism, dysregulated Ca^2+^ buffering, compromised iron transport, and changes in mitochondrial dynamics, and these could translate into insufficient ATP production, increased ROS, and cell death. Mitochondrial dysfunctions can be secondary events or consequences of altered metabolic pathways: the cause of inflammation affecting adipose tissue in obesity is not clear, but it is known that mitochondria are important for maintaining metabolic homeostasis in white adipocytes [28, 29], and it is known that an excessive substrate availability affects mitochondrial functions [30–32]. Dyslipidemia and increased concentration of long-chain fatty acids could induce oxidative stress and mitochondrial dysfunction [33]; increased production of toxic A*β* oligomers has been shown to impair mitochondria in AD [34]. But mitochondrial dysfunctions could represent early events in the development of a pathology: in a number of studies, mitochondrial inheritance has been associated with obesity and type 2 diabetes, as well as metabolic syndrome, insulin resistance, and cardiovascular disease [35–37]. A recent study has found an interesting genetic association of mitochondrial, nuclear mitochondrial variants and genes with seven metabolic traits comprising body mass index, waist-hip-ratio, fasting glucose, fasting insulin [38]: traits characteristic of cardiovascular disorders, obesity, diabetes, and neurodegeneration. These findings point to a subtler role of the mitochondria in the development and possible origin of complex metabolic disorders and make worth investigation of possible connections among the mitochondria, metabolism, and complex metabolic disorders.

## 2. Mitochondria Structure and Functions

**Figure 1:**
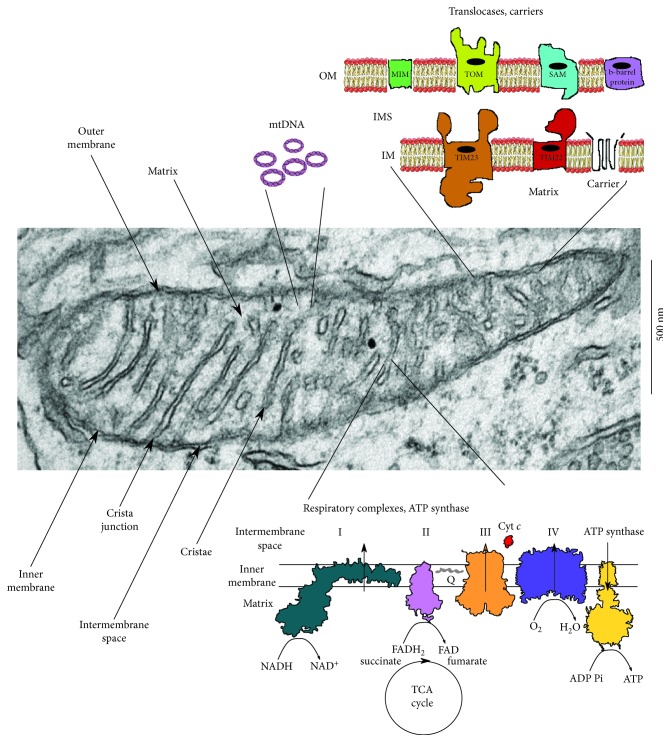
Electron micrograph of mouse brain mitochondria, cartoons of some mitochondrial structures, and their localization within the organelles.

Mitochondria originated from the endosymbiosis established between an alpha-proteobacterium and a prokaryote cell [39–41] that took place only once in eukaryotic evolution [42]. It is commonly believed that the origin of complexity preceded the acquisition of mitochondria [43, 44], but recently, it has been proposed that mitochondria are prerequisite to complexity [45], because oxidative phosphorylation across the wide area of the internal membrane enabled an almost 200,000 fold expansion of the original cellular genome size. The energy necessary to sustain the expression and the working of such a large number of proteins is provided mostly by the mitochondria that, for this purpose, retain a core of genes required for the expression of proteins of the respiratory electron transport chain and of the ATP synthase [46]. The rest of about 1200 proteins present in the mitochondria are encoded by genes present in the nucleus. This peculiar feature makes the mitochondria, and the cell as well, dependent on the coordinated cross talk between two independent genomes, the nuclear and the mitochondrial one, increasing the possibilities of malfunctioning and, for this reason, demanding layers of checkpoint controls. In addition to providing energy for the cell, the mitochondria are essential for signal transduction, cell proliferation, and cell death; this array of important functions makes the regulation of mitochondrial biogenesis vital for proper cellular functioning [47]. The damage of normal mitochondrial dynamics caused by mutations or environmental toxins can result in homeostatic imbalances that can hamper mitochondrial membrane potential and all the functions relying on it, such as ATP production and active transport of proteins and ions across the membrane; also, the fall in energy charge can result in an increase in free radical production and altered mitophagy [48, 49]. All these events can have direct impact in the development and progression of complex disorders.  Figure 1 shows a representative image of detailed mitochondrial morphology.

## 3. Membranes and Membrane-Delimited Domains

Mitochondria possess two separate and functionally distinct membranes, the outer membrane (OM), which separates the inside of the organelle from the rest of the cell and provides the platform for the communication interaction of the mitochondria with other cellular compartments, and the ultra-specialized and highly folded inner membrane (IM). The building blocks of the two membranes are represented mostly by phospholipids, while sterol content is usually low, with the exception of the cells that synthetize steroid hormones [50, 51]. The most abundant phospholipids are phosphatidylcholine (PC) and phosphatidylethanolamine (PE), representing respectively about 40 and 30 percent of total phospholipids; the less represented are phosphatidylinositol (PI) and phosphatidylserine (PS), together comprising about 6 percent of total phospholipids [52, 53]. Interestingly, the lipid composition of the OM differs significantly from that of the inner membrane [54, 55]; moreover, phospholipid distribution is not even across the leaflets of the membranes [56, 57]. Both these features point to a specific physiological role for each class of phospholipids. Cardiolipin (CL) is the signature lipid of the mitochondria, with content in the range of 15-20 percent, and it is enriched in the IM [58, 59]; it regulates mitochondria bioenergetics by binding to and influencing the activity of respiratory chain complexes [60, 61]; furthermore, it also stabilizes the respiratory supercomplexes [62, 63]. Consistent with this function is the observation that CL-deficient mitochondria have a decreased membrane potential [61]. Cardiolipin, as well as cardiolipin remodeling [64, 65], is fundamental for the structure of the inner membrane and is critical for membrane dynamics, both IM fusion [66, 67] and fission [68, 69], via its interaction with mitochondrial proteins OPA1 and Drp1, respectively. In addition, CL has an important role in mitochondria quality control: the externalization of CL to the outer membrane is recognized as a signal for mitophagy, likely through a specific interaction with microtubule-associated protein 1A/1B-light chain 3 (LC3) [70, 71].

The outer membrane is critical for the communication and interactions between the mitochondria and the rest of the cell; the passage of small solutes and larger molecules is finely regulated by several channels. Ions and small molecules exchange through the abundant voltage-dependent anion channel (VDAC) [72]. Bigger molecules, especially proteins, are imported via specialized channels, part of large translocase complexes [73]. Tom40, the main protein import channel of the translocase of the outer membrane (TOM), transports numerous proteins presenting positively charged targeting sequences [74]; Sam50 of the sorting and assembly machinery (SAM) inserts *β*-barrel precursor proteins into the outer membrane [75, 76]. Yeasts also possess Mdm10, the mitochondrial distribution and morphology protein which, together with its role in the sorting and assembly machinery [77], acts as a membrane anchor of the ER-mitochondrial encounter structure ERMES tethering the ER and mitochondria [78, 79]. Recently, other proteins forming non-*β*-barrel channels on the mitochondria outer membrane have been discovered in yeast, suggesting a higher than expected degree of fluxes' regulation at the level of the OM [80].

The IM presents a much more expanded surface than the surrounding OM, because of its largely folded structure. Given its characteristic conformation, it is considered to be composed by two morphologically identifiable subdomains: the portion of the membrane closely apposed to the outer membrane is called the inner boundary domain, while the tubular or lamellar structures that extend into the central mitochondrial compartment are the cristae [81]. The inner boundary membrane is enriched with proteins participating in the transport of ions (i.e., the Ca^2+^ uniporter; the Ca^2+^/Na^+^ exchanger), metabolites (pyruvate carrier, ATP/ADP exchanger), and proteins (the two major complexes are TIM22, the carrier translocase of the inner mitochondrial membrane, and TIM23, the presequence translocase of the inner mitochondrial membrane) [82–84].

The cristae membranes are the main site where oxidative phosphorylation takes place, as they host the fully assembled complexes of the electron transport chain and the ATP synthase. Cristae architecture is quite heterogeneous, depending on the cell type and the energetic state of the mitochondria [85–87].

Cristae are connected to the inner boundary domain by narrow tubular structures of varying length and diameter ranging from 12 to 40 nm: the crista junctions [88–90]. The hallmark shape of the crista junctions characterized by a high negative curvature of the membrane is produced and maintained by a conserved mitochondrial contact site and crista organizing system (MICOS). Two distinct MICOS subcomplexes have been identified: one, marked by the core component Mic60, is directly involved in the formation of contact sites between the outer and the inner membrane and is thought to stabilize crista junctions via interaction with the outer membrane TOM and SAM complexes [91, 92]. The second MICOS subcomplex is characterized by the presence of the Mic10 core component which has the ability to oligomerize and bend the IM and is critical for the formation of crista junctions [93, 94].

The crista junctions are thought to represent a barrier for the free movement of soluble proteins and metabolites between the intermembrane and the intracrista space [89].

The intermembrane space (IMS) between the inner and outer membranes possesses an ionic composition similar to the one of the cytosol, but a specific protein content [95]. Some physicochemical properties of the IMS have been reported to differ from the ones of the cytosol. For example, the pH, measured with targeted pH-sensitive GFP, is proved to be slightly more acidic [96]; also, the ratio between reduced and oxidized glutathione is lower, and the IMS compartment at the steady state is more oxidizing than the cytosol [97]. The many functions of this compartment are being defined, clarifying its critical role in the coordination of mitochondrial activities with several cellular processes including the exchange of proteins, lipids, and metal ions between the matrix and the cytosol; the mediation of signaling pathways that regulate respiration and metabolic functions; the regulated initiation of apoptotic cascades; the confinement of reactive oxygen species produced by the respiratory chain; or the control of mitochondrial morphogenesis [98]. The constriction represented by the crista junctions limits the free diffusion of molecules within the IMS and separates this space into two discrete regions. This compartmentalization could serve to optimize mitochondrial performance: the restricted diffusion of protons might improve the efficiency of the respiration-driven ATP synthesis. Also, large amounts of the soluble electron carrier protein cytochrome *c* are confined within the lumen of the cristae in close proximity to the position where respiratory chain complexes are preferentially found [99, 100], and the release of cytochrome *c* from the crista space during the apoptotic process requires opening of the crista junctions [101, 102].

The inner membrane surrounds the innermost mitochondrial compartment: the matrix which is the site of mitochondrial DNA (mtDNA) replication and transcription, of protein synthesis, and where numerous enzymatic reactions take place. These biosynthetic reactions include those of the tricarboxylic acid cycle (TCA) and fatty acid *β*-oxidation. The pH of the matrix is kept at around 8 [103], slightly higher than the cytoplasmic pH which is around 7 and higher than the intermembrane pH: this difference in proton concentration is exploited by the ATP synthase to drive the synthesis of the high energy adenosine triphosphate (ATP) molecule.

## 4. Respiratory Chain

The inner membrane hosts the four large complexes of respiratory chains I-IV and the ATP synthase. Complex I, NADH/ubiquinone oxidoreductase, is the largest respiratory complex; the bovine enzyme contains at least 45 subunits and has a molecular mass greater than 900 kDa [104]. It represents the entry point of the electrons (e^−^) in the respiratory chain: two e^−^ donated by NADH are used to reduce membrane soluble ubiquinone to ubiquinol, and the process is coupled with the vectorial transport of four protons across the IMM. So the energy released in the transfer of electrons through complex I, and also complexes III and IV, is not lost to heat; instead, it is used to pump protons across the membrane to build the mitochondrial membrane potential. Complex I consists of two domains, also called “arms”: the hydrophobic arm is inserted into the membrane, while the hydrophilic arm protrudes into the matrix. The minimal module of complex I sufficient to catalyze energy transduction is composed of 14 core subunits. In eukaryotes, the 7 core subunits of the membrane arm are encoded by the mitochondrial genome, while the 7 core subunits of the peripheral arm are encoded by the nuclear genome. In addition to the core subunits, complex I of the eukaryotes possesses a large number of accessory subunits [105, 106]. The reaction of mitochondrial complex I is reversible, and e^−^ can be transferred from ubiquinol back to NAD^+^ at the expenses of the proton motive force. This reaction can cause an increase in superoxide production, which is rapidly dismutated into hydrogen peroxide H_2_O_2_ [107]. An interesting feature of complex I is that it can assume two different states: active state A and dormant state D [108], characterized by structural rearrangements and different catalytic activities [109, 110]. The A-form is predominant during steady-state aerobic respiration; it catalyzes the fast NADH:ubiquinone oxidoreductase reaction. In low-oxygen conditions, the A-form can convert to the D-form which has a lower activity and an initial lag phase; the D-form can be reactivated in the presence of O_2_ and ubiquinone. The A/D transition during ischemia reperfusion episodes has the ability to limit the “burst” of ROS which occurs in the early stage of reperfusion when O_2_ levels increase and metabolic intermediates are imbalanced [111].

In the mammalian mitochondria, complex II succinate dehydrogenase (SDH) directly reduces coenzyme Q with e^−^ donated by the FADH coenzyme. It is both a component of the TCA cycle (it catalyzes the oxidation of succinate to fumarate) and an electron transfer complex, and it is the only component of the respiratory chain encoded exclusively by nuclear genes (only few exceptions are known [112]). It is composed of a flavoprotein which contains the FAD cofactor and an iron-sulfur protein which has three FeS clusters: these form the catalytic subunit; in addition, mammalian complex II has two transmembrane proteins (SDHC and SDHD) [113] that contribute the ubiquinone binding sites. At variance from the other complexes of the respiratory chain, complex II does not pump protons across the IMM. As SDH lies at the crossroad of two essential pathways, emerging evidences show that both its biosynthesis and function are closely regulated at posttranslational levels to adapt to metabolic demands [114], and its altered regulation has been linked to several diseases [115, 116].

Complex III, cytochrome *c* reductase, comprises 11 subunits; it accepts e^−^ from reduced coenzyme Q (ubiquinol) and transfer them to cytochrome *c*. It is a symmetrical dimer with three conserved core subunits: cytochrome *b* (cyt b), cytochrome *c*_1_ (cyt *c*_1_), and the Rieske iron-sulfur protein [117]. cyt *b* is the only mtDNA-encoded subunit of complex III; it provides the complex with two distinct binding sites for quinone [118]; while the soluble domain of cyt *c*_1_ provides the site of interaction with cytochrome *c* [119]. Complex III is another site of ROS production, and it can possibly release ROS both in the IMS and in the matrix [120]. ROS produced by complex III are thought to have a signaling role contributing to promote apoptosis [121] or to produce the adaptive response to hypoxia [122].

Complex IV, the mitochondrial cytochrome *c* oxidase (COX), is the terminal oxidase of the mitochondrial respiratory chain: it catalyzes the transfer of electrons from reduced cytochrome *c* to O_2_, coupling it to the pumping of protons to the intermembrane space. It is a multimeric enzyme composed of three subunits (COX1-3) conserved from *α*-proteobacteria; these subunits are encoded by the mtDNA and form the catalytic core of the complex [123]. In mammalian cells, up to 11 more subunits encoded by the nuclear genome participate in the COX structure and regulate the enzyme activity to coordinate energy production with the cellular needs [124]. The nuclear encoded subunits present multiple tissue-specific isoforms with slightly different structural features that could impinge on the organization of the holocomplex and on the performance of the enzyme, perhaps helping to meet the different metabolic needs of the different tissues [125, 126]. Impaired COX functioning has consequences on cellular energy metabolism and could result in increased ROS production.

## 5. Supercomplexes

The respiratory chain complexes interact with various stoichiometries to form supercomplexes [127, 128]. The assembly of one unit of complex I, two units of complex III, and one of complex IV is often called the respirasome. The supramolecular association stabilizes individual complex and is thought to increase the efficiency of direct electron transfer from the donor to the acceptor complex by reduction of the diffusion time [129, 130]; it may also decrease the amount of ROS produced during electron transport [131, 132]; the association between complex I and complex III might contribute to sequestering ubisemiquinone, the reactive ubiquinol intermediate which can react with O_2_ to generate superoxide.

## 6. F_1_-F_o_ ATP Synthase

The F_1_-F_o_ ATP synthase is a complex nanomachine that uses the electrochemical proton gradient generated by the respiratory chain to synthesize ATP. It comprises two functional domains: the soluble portion, F_1_ situated in the matrix, and the F_o_ part embedded in the IMM. F_1_ is composed of three copies of each subunit *α* and *β*, plus one copy of the subunits *γ*, *δ*, and *ε*; these last components form the central stalk of the complex. F_o_ consists of 8 copies (in bovine mitochondria) of protein c forming a ring structure [133]. One copy of each subunit a, b, d, F6, and OSCP forms the peripheral stalk which lies on one side of the complex. Additional membrane subunits e, f, g, and A6L are associated with F_o_ [134, 135]. Only subunits a and A6L are encoded by the mitochondrial genome [136]. Protons move through the F_o_ complex embedded in the membrane, driving the motion of the rotor composed of the ring of c subunits and of *γ*. The central stalk propagates the momentum to the catalytic F_1_ subunit where ADP and phosphate react, through a sequence of conformational changes, and F_1_ catalyzes the synthesis of ATP. When the respiratory chain is impaired or cannot sustain the potential difference across the IMM (Δ*ψ*), the F_1_F_o_ ATP synthase can work in reverse hydrolyzing ATP and pumping protons from the matrix to the IMS [137]. The ATP synthase associates in dimers via the F_o_ portion of the holocomplex [138, 139], and the dimers have been shown to form double rows along the crista edges of the IMM. Molecular dynamics simulations suggest that the dimers are able to bend the lipid bilayer and contribute to form the cristae [140]. Recently, a novel function has been proposed for the ATP synthase as the core component of the mitochondrial permeability transition pore (PTP), allowing the regulated opening on the inner membrane of a channel with a molecular cut-off of about 1500 Da [141].

## 7. Mitochondrial DNA

Mitochondria possess their own DNA (mtDNA) with a specific genetic code distinct from the nuclear one. mtDNA is a circular molecule of 16.5-kilobase (kb) pairs, compacted into DNA-protein supramolecular assemblies called the nucleoids with a diameter of about 100 nm and present at approximately 1000 copies per cell [142, 143]. At variance from the nuclear DNA, mtDNA has mostly a uniparental inheritance, and for the majority of organisms examined, it is inherited from the female parent [144]. The mtDNA has been reduced during evolution with the transfer of about 99 percent of its genes to the nucleus; it encodes the core genes for bioenergetics: 13 polypeptides of the respiratory chain in complexes I, III, and IV, and in the ATP synthase; two ribosomal RNAs (mt-rRNAs); and 22 transfer RNAs (mt-tRNAs). All these genes are essential for normal mitochondrial function. In addition, there is a noncoding sequence, the displacement loop (*D-loop*), which comprises the replication origins and promoters for mtDNA. Mitochondria can respond to changes in membrane potential modulating the expression of respiratory chain proteins [145, 146].

Respiration rate correlates with the amount of mtDNA in the cells [147], and precise regulation of the mtDNA copy number is necessary for maintaining cellular energy demands: cells with high energy needs, such as neurons and muscle cells, are mostly at risk of energetic failure as a consequence of mtDNA mutation/depletion; in fact, mtDNA depletion or increased degradation has been implicated in human diseases such as Parkinson's [148, 149], Alzheimer's disease [150, 151], and aging [152, 153]. Compared to the nuclear DNA, mtDNA has a much high sequence evolution rate [154, 155] that could result in the generation of numerous mutations throughout the life of the cell and the entire organism, and mtDNA variants could influence individual susceptibility to complex diseases [156, 157].

## 8. Alzheimer's Disease

Among neurodegenerative disorders, Alzheimer's disease (AD) is the most common form of dementia worldwide [158]; it is characterized by a progressive and fully debilitating decline in cognitive functions. Fundamental neuropathological hallmarks of AD are the extracellular deposits of insoluble proteins, the plaques, primarily composed of amyloid-*β* (A*β*) peptides, and the intracellular neurofibrillary tangles (NFTs) mainly constituted by phosphorylated microtubule-associated protein tau (Mapt). Compared to nonaffected subjects, tau is abnormally phosphorylated in the brain of AD patients and this can contribute to its missorting to dendritic filaments and to its polymerization and aggregation [159]. A correspondence is observed between NFT deposition and synapse loss during the course of AD, and each of these processes further correlates with cognitive decline [160].

The large majority of AD cases are sporadic with age being the single most important risk factor, and the presence of the *ε*4 allele of apolipoprotein E a substantial contributing element [161]. A small, but significant, percentage of cases (between 1 and 5 percent), called collectively familial Alzheimer's disease (FAD), is due to dominant mutations in one of the three genes involved in the synthesis and processing of the amyloid protein. These genes encode for the amyloid precursor protein (APP), presenilin 1 (PS1), and presenilin 2 (PS2), respectively, and the pathological mutations cause an aberrant production of A*β* peptides: soluble A*β* peptides of various lengths are thought to be the most pathological forms contributing in the dysfunction and ultimately the demise of neurons. The substantial pathological identity between the sporadic and the familial forms of the disease contributed to the proposition of A*β* deposition as the central event in the genesis of the disease (the amyloid cascade hypothesis (ACH)) [162, 163]; though developing, increased knowledge of the disorder posits that this hypothesis falls short of explaining its complete etiology [164]. In fact, the precise origin(s) of the disease is not completely understood, but it is now thought that about 70 percent of the AD risk is attributable to genetic factors: in fact, a combination of environmental and genetic elements most likely contributes to the disorder though the pathways involved are incompletely understood [165]. Thus, AD appears as a very complex process, perhaps consisting of different interacting phases, characterized by the individual contribution, as well as the interaction, among the different cell types present in the affected brain areas [166].

## 9. Obesity

Obesity is a metabolic disorder that causes an abnormal or excessive fat accumulation that can severely impair health. The fundamental cause of obesity is the energy imbalance between calories consumed and calories expended, as the result of combined increased intake of energy-rich food, and the decrease in physical activity. Obesity is a major risk factor for several other diseases such as diabetes and cardiovascular diseases and some types of cancer [167]. It is characterized by low-grade, chronic inflammation [16], an increase in oxidative stress [168, 169], and also, mitochondrial dysfunction and altered mitochondrial biogenesis have been frequently observed [170–172]. It is still not known how mitochondrial functioning changes in the adipose tissue in obesity, whether it contributes to the early development of the metabolic alteration and whether it is genetic or acquired, but mitochondrial dysfunction has been implicated in the development of insulin resistance [173–175] which is a common feature in obesity.

## 10. Diabetes

Diabetes comprises an early onset (already in early childhood) disease denominated type 1 caused by the complete failure of the pancreatic islet *β*-cell to produce insulin, and it requires exogenous insulin administration for survival; and a chronic, progressive disease denominated type 2 (T2D) characterized by an initial increased insulin secretion leading to islet stress and loss of glucagon/insulin homeostasis. Both disorders present an altered glucose homeostasis that can have serious adverse outcomes compromising brain functions and altering the functionality of peripheral tissues. T2D is a complex disorder resulting from the interaction between genes and the environment, and several risk factors have been identified ranging from age to sex, diet, and ethnicity. It often associates with obesity and insulin resistance. Genetics is an important factor in the development of T2D, though heritability estimates are quite variable among different studies, from 22 to 69 percent, and might depend on the age of onset [176, 177]. The challenge is to find genetic markers that explain the increased risk associated with a family history of diabetes. T2D is characterized by an increase in proinflammatory cytokines and chemokines that involves several organs, and the characteristic hyperglycemia results in amyloid deposits that cause increased redox stress for the cells [178].

T2D is considered a protein misfolding disorder associated with the accumulation of islet amyloid polypeptide (IAPP) in pancreatic islets. IAPP, also called amylin, is a peptide hormone cosecreted with insulin from the pancreas. Amylin is elevated in the blood of T2D patients, and misfolded IAPP has been found in the brain of AD patients with T2D [179]; also, A*β* and phosphorylated tau can be found in the pancreas of T2D patients [180]. T2D and impaired fasting glucose represent a comorbidity in about 80 percent of AD patients [181].

**Figure 2:**
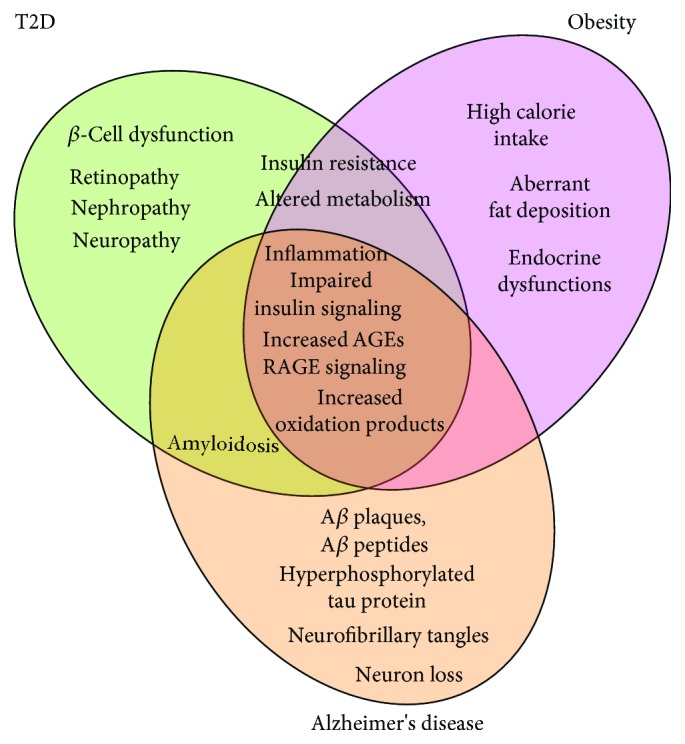
Specific and common features among type 2 diabetes, obesity, and Alzheimer's disease.

Disease-specific, and common phenotypes among T2D, obesity, and Alzheimer's disease are illustrated in Figure 2.

## 11. Association between Obesity, Diabetes, and AD

Several common medical conditions such as heart disease, type 2 diabetes, obesity, and late onset neurodegenerative disorders do not have a single genetic cause: they are likely associated with the effects of multiple genes (polygenic) in combination with lifestyle and environmental factors. These multifactorial disorders often cluster in families, but do not have a clear pattern of inheritance, and this makes it difficult to determine a person's chance of inheriting or passing on these disorders.

Recently, substantial evidence points to a link between metabolic diseases such as obesity and diabetes and a person's susceptibility to develop Alzheimer's disease. In particular, several longitudinal studies observed an association between a larger waist-hip ratio in middle-age and the increased risk of developing dementia later in life [182] or with a decreased hippocampal volume [183]. Also, a significant relation between obesity in midlife, and the risk of developing dementia later in life has emerged in several epidemiological studies [184–188]. It is worth mentioning, though, that a retrospective cohort study has observed an increased risk of dementia in later life associated with underweight in middle age, questioning the link between obesity and dementia [189]. So these correlations between obesity and dementia are worth more to be studied in order to dissipate discrepancies. High glucose levels have also been found to be a risk factor for dementia, even among people without diabetes [2], and high levels of glycosylated hemoglobin have been associated with lower scores in cognitive function tests [190–192]. A mechanistic link between obesity, diabetes, and dementia could be the low-grade peripheral inflammation, due to chronically elevated levels of circulating free fatty acids, and the development of insulin resistance [193, 194]. Insulin resistance, defined as an insufficient cellular response to a given dose of the hormone, decreases the ability of the cell to preserve energy homeostasis. In the brain, proinflammatory molecules such as TNF*α* released from activated microglia were found to contribute to impaired insulin signaling and synapse loss via aberrant activation of JNK signaling that in turn causes a decreased activation of PI3-kinase and Akt proteins with consequences for cellular growth and increased susceptibility to apoptosis.

Increased inflammatory signaling leads also to an increase in ROS production. In the brain, controlled ROS production is involved in synaptic signaling and mechanisms related to long-term potentiation and memory consolidation [195]; an imbalance between ROS production and antioxidant defenses [196] could lead to oxidative stress and increased amounts of oxidized lipids, proteins, and nucleic acids [197]. In fact, in the AD brain, an augmented ROS production occurs together with suppressed mitochondrial fast axonal transport, [198] fragmentation of neuronal mitochondria and impaired mitochondrial dynamics at synapses [199].

A subtle but chronic impairment in energy production could also develop in neurodegeneration later in life. More recently, an early developing metabolic defect impinging most likely on glycolysis and resulting, as a cascade effect, also on mitochondria energy production with a significant decrease in NAD(P)H concentration has been observed in a mouse model of familial AD disease bearing a mutated form of human PS2 and mutated human APP [200].

## 12. Mitochondria and Metabolic Disorders

Mitochondria might not be simply the subjects on which altered metabolism impinges causing their malfunctioning but could also be principal actors conditioning the cellular/organismal response to the environment. Dysregulated mitochondrial Ca^2+^ signaling and Ca^2+^ homeostasis are involved in the pathogenesis of insulin insensitivity and T2D [201], and altered ER mitochondrial Ca^2+^ signaling is a crucial aspect in the impairments produced by the mutated PS2 protein that can have a major role in the development of neurodegeneration in FAD cases [202, 203].

The presence in the cell of two distinct genomes implies that the nucleus and mitochondria must coordinate transcription and translation and also the translocation of mitochondrial proteins [204]. The nucleus controls mitochondrial functions through so-called “anterograde regulation,” modulating mitochondrial activity and promoting mitochondrial biogenesis to meet the cellular needs. Conversely, mitochondria signal to the nucleus the need to modify nuclear gene expression for adaptive cellular responses via “retrograde response” [205]. Miscommunication between the nucleus and mitochondria can have important consequences for the cell; defects in the regulation/expression of several mitochondrial proteins have been associated with metabolic phenotypes in animal models and in humans. Altered functioning of several mitochondrial chaperones HSP60, HSP90, and HSP72 has resulted closely associated with insulin resistance, obesity, and type 2 diabetes, both in animal models and in humans [206–208]. Mitochondria also possess an array of conserved proteases important for quality control: the LON protease (LONP1) degrades oxidized proteins within the matrix, and a reduced expression has been associated with chronic stress, aging, and age-related disorders [209]. The protease presenilin-associated rhomboid-like (PARL) regulates oxidative phosphorylation in skeletal muscle and insulin signaling; knockdown of PARL causes malformation of the cristae, increases oxidative stress, and impairs insulin signaling [210].

The mitochondrial genome itself has been shown to deeply influence several aspects of physiology ranging from insulin signaling and obesity to telomere shortening and median lifespan: mtDNA haplotypes, nonpathological mitochondrial DNA variants, appear to have a broad effect on metabolism, and the differences observed seem to be more pronounced later in life [211].

The dual genomic origin of mitochondrial components, from mtDNA and nDNA, with the former subjected to a high mutational rate [154, 155] prompted the hypothesis that mitochondrial variants influence individual susceptibility to complex diseases [156, 157]. In particular, human mtDNA presents large sequence variability within populations, and many studies have suggested that mtDNA variants may be associated with ageing or diseases. Moreover, a generalized, systemic energy defect could in fact result in organ-specific symptoms since mitochondrial dysfunctions affect the cellular energy balance [212], and energy demands differ between different cell types and different organs.

A recent study has associated mitochondrial and nuclear mitochondrial variants with seven metabolic traits ranging from body mass index (BMI) and glycosylated hemoglobin (HbA1c) to fasting glucose and insulin [38]. The study examined risk factors involved mainly in cardiovascular disease, but traits have emerged linked to some neurodegenerative disorders and in particular to Alzheimer's disease. In fact, a number of these variants are also found among the risk genes for Alzheimer's disease discovered by genetic meta-analysis of clinically diagnosed late-onset Alzheimer's disease [213]; common risk genes are summarized in Table 1.

## 13. Conclusions

A growing body of data points to a close relationship between metabolic disorders such as obesity, diabetes, and neurodegeneration. Moreover, altered mitochondrial functions emerge in all those diseases. Perhaps, this comes not as a surprise given the numerous, important functions sustained by the mitochondria in the cell: in particular the production of energy necessary for all cellular functions and also the release of signaling molecules in response to external stimuli. All these aspects are worth investigating for a better insight of chronic and debilitating disorders that do not have a univocal etiology and that are affecting an ever-increasing number of people worldwide. In particular, a deeper understanding of the interaction of a specific mtDNA haplotype and the environment could provide the development of preventative strategies to delay or hopefully block the progression of the disorders.

## Figures and Tables

**Table 1:** Common risk genes identified in genetic meta-analysis of Alzheimer's disease and in the study of metabolic traits associated with mitochondrial and nuclear mitochondrial variants and genes [38].

Gene SNV	Full gene name	SNV
WWOX	WW domain-containing oxidoreductase	rs1847591
MTCH2	Mitochondrial carrier 2	rs4752856
PSMC3	Proteasome 26S subunit, ATPase 3	Expression regulated by MTCH2 rs4752856
C1QTNF4	C1q and TNF-related 4	Expression regulated by MTCH2 rs3817335
CELF1	CUGBP Elav-like family member 1	Expression regulated by MTCH2 rs7118178
ACP2	Acid phosphatase 2, lysosomal	Expression regulated by NR1H3 rs7120118
